# General practitioner strategies for managing patients with multimorbidity: a systematic review and thematic synthesis of qualitative research

**DOI:** 10.1186/s12875-020-01197-8

**Published:** 2020-07-01

**Authors:** Raechel A. Damarell, Deidre D. Morgan, Jennifer J. Tieman

**Affiliations:** Research Centre for Palliative Care, Death and Dying, College of Nursing and Health Sciences, Flinders University, GPO Box 2100, Adelaide, South Australia 5001

**Keywords:** Multimorbidity, General practice, Patient-centred care, Evidence-based practice, Qualitative, Meta-synthesis

## Abstract

**Background:**

General practitioners (GPs) increasingly manage patients with multimorbidity but report challenges in doing so. Patients describe poor experiences with health care systems that treat each of their health conditions separately, resulting in fragmented, uncoordinated care. For GPs to provide the patient-centred, coordinated care patients need and want, research agendas and health system structures and policies will need to adapt to address this epidemiologic transition. This systematic review seeks to understand if and how multimorbidity impacts on the work of GPs, the strategies they employ to manage challenges, and what they believe still needs addressing to ensure quality patient care.

**Methods:**

Systematic review and thematic synthesis of qualitative studies reporting GP experiences of managing patients with multimorbidity. The search included nine major databases, grey literature sources, Google and Google Scholar, a hand search of *Journal of Comorbidity*, and the reference lists of included studies.

**Results:**

Thirty-three studies from fourteen countries were included. Three major challenges were identified: practising without supportive evidence; working within a fragmented health care system whose policies and structures remain organised around single condition care and specialisation; and the clinical uncertainty associated with multimorbidity complexity and general practitioner perceptions of decisional risk. GPs revealed three approaches to mitigating these challenges: prioritising patient-centredness and relational continuity; relying on knowledge of patient preferences and unique circumstances to individualise care; and structuring the consultation to create a sense of time and minimise patient risk.

**Conclusions:**

GPs described an ongoing tension between applying single condition guidelines to patients with multimorbidity as security against uncertainty or penalty, and potentially causing patients harm. Above all, they chose to prioritise their long-term relationships for the numerous gains this brought such as mutual trust, deeper insight into a patient’s unique circumstances, and useable knowledge of each individual’s capacity for the work of illness and goals for life. GPs described a need for better multimorbidity management guidance. Perhaps more than this, they require policies and models of practice that provide remunerated time and space for nurturing trustful therapeutic partnerships.

## Background

Primary care providers and the systems in which they operate are increasingly called upon to manage patients with two or more co-occurring chronic medical conditions, or ‘multimorbidity’ [1]. This epidemiological shift has been attributed to the greater longevity offered by improvements in therapeutic technologies along with the increased risks associated with unhealthy lifestyles [1, 2]. One systematic review has estimated prevalence in general practice to be 12.9% for adults and 95.1% in a community-dwelling population aged 85 years and older [3]. While much of this variance can be attributed to inconsistencies in the way multimorbidity is defined and measured across studies, it nevertheless points to a significant problem that rises sharply with age.

Multimorbidity appears to be socially patterned, appearing more frequently [3] and 10 to 15 years earlier in populations living in areas of socioeconomic deprivation [4]. Furthermore, simulation modelling based on current risk factors estimates a two-fold increase in the prevalence of complex multimorbidity (four or more conditions) by 2035 [5].

Multimorbidity impacts on patient quality of life in significant ways [6–8]. Conditions might impart a high symptom burden [9] while their treatments can result in adverse side effects or inappropriate polypharmacy [10]. Functional or cognitive decline leading to reduced autonomy might also impact on an individual’s psychosocial health [11] and sense of life purpose [12, 13] and some long-term progressive conditions, such as heart failure and chronic obstructive pulmonary disease (COPD), reduce life expectancy [14]. Patients with multimorbidity are often heavy users of health care, frequently traversing primary and secondary care sectors to visit a range of specialists, each focused on a particular condition or body system in isolation [15]. This siloed model can leave patients struggling to harmonise and adhere to complex medication regimens [16, 17]. When multiple appointments and therapeutics are added to the challenges presented by their illnesses, patients and their families/carers may experience an excessive burden of treatment [18, 19]. At times, this burden may exceed the patient’s capacity to do the ‘work’ being asked of them.

Patients with multimorbidity desire care which is less fragmented and better coordinated across the system [20, 21]. General practice may be best suited to take an increased share of responsibility for coordinating care across sectors being based on ‘longitudinal continuity of care as determined by the needs of the patient’ [22]. Patients with multimorbidity are already high users of general practice in countries such as England and Australia where the general practitioner (GP) acts as gatekeeper to other health specialists [23, 24]. In many places, however, models of general practice may remain structured around single disease management, reflecting the traditional approach that still dominates secondary care, medical education curricula, and the research agenda behind the production of the evidence that informs clinical practice [4].

**Fig. 1:**
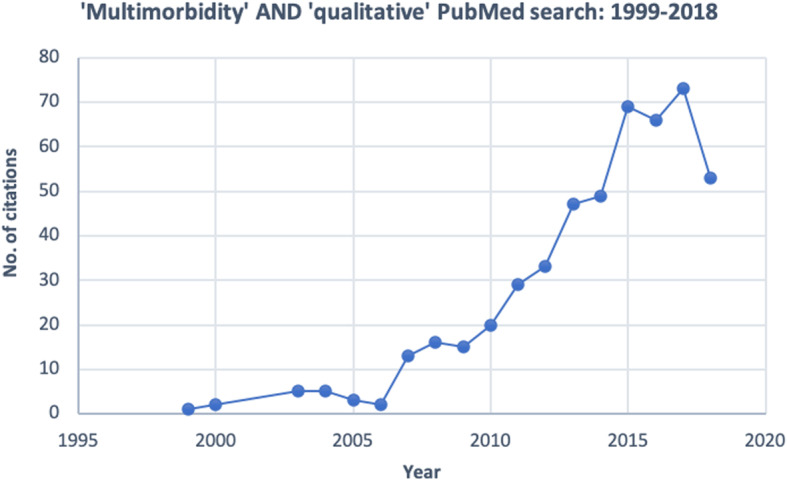
Growth in qualitative multimorbidity research literature: 1999–2018

A 2013 systematic review of GP experiences in managing multimorbidity revealed several challenges to care provision including the inadequacy of the evidence base for multiple chronic conditions and the prevailing structure of the primary health care system [25]. Since this review, awareness of multimorbidity and its impact on patients and health care systems has grown with the Academy of Medical Sciences labelling it ‘a priority for global health research’ [1]. New primary care models for managing multimorbidity are being discussed and trialled, such as the patient-centred 3D study in the United Kingdom [26] and the Australian government’s Health Care Homes pilot [27]. Furthermore, organisations such as National Institute for Health and Care Excellence (NICE) and the American Geriatrics Society (AGS) have produced multimorbidity guidelines in the form of general guiding principles of care [28, 29]. The research literature on multimorbidity has also increased exponentially in this time [30], including the number of primary qualitative studies investigating GP perspectives. (See Fig. 1.)

For these reasons, this systematic review seeks to build on the 2013 review by asking whether the challenges put forward by GPs in the literature prior to 2013 remain the same today. Moreover, do GPs adapt their approach to care when managing patients with multimorbidity, and are these approaches working?

Rather than create a new, more recent review to complement the first, we chose to integrate studies from the previous synthesis with any newer studies to produce a more convenient single set of findings. This ‘knock the house down and rebuild it’ approach [31] also allowed us to use a different search strategy, broader inclusion criteria and a different method of synthesis to the original review. It also avoided drawing an arbitrary dividing line between the two reviews, the first of which only included two years’ worth of studies (2010–2012). This systematic review is reported according to the ENTREQ framework [32].

### Objectives

This systematic review aims to understand if and how multimorbidity impacts on the work of general practitioners by analysing the collective firsthand data within existing qualitative primary studies. It also seeks to identify GP strategies and proposed solutions for dealing with challenges to ensure quality care provision.

The review questions are therefore:

- Which aspects of patient care are considered challenging for GPs in the therapeutic management of patients with multimorbidity?
- What strategies do GPs engage to handle these challenges in order to provide satisfactory patient care?
- What changes do GPs believe necessary to improve the care of patients with multimorbidity and their own experiences?

## Methods

Qualitative studies were deemed best suited to answering the review questions as their methods provide ‘an approach for exploring and understanding the meaning individuals and groups ascribe to a social or human problem.’ [33] To synthesise this qualitative research data we employed the ‘thematic synthesis’ methodology of Thomas and Harden [34]. We chose this approach for several reasons. Firstly, we anticipated having to synthesise a large number of studies and this methodology is considered better suited to that purpose than other methodologies [32, 35]. Secondly, this approach does not integrate data merely to quantify the prevalence of certain concepts. The integrated data undergoes interpretation which can lead to new, novel insights on an issue [36]. Thirdly, thematic synthesis provides a systematic and transparent approach to conducting and reporting the review through its three clearly delineated stages. These stages are line-by-line inductive coding of findings within the primary studies; organising any related ‘codes’ into descriptive ‘themes’; and the creation of more abstract ‘analytic themes’ [34].

### Search strategy

We used a diverse range of search strategies in the interests of comprehensive retrieval for ‘conceptual saturation’ and ‘maximal variability in findings’ [34], as well as to counter known challenges in identifying qualitative research using electronic databases [37, 38].

A database search strategy was first developed and tested in the Ovid Medline database. This comprised a combination of database subject headings and free text terms for three distinct concepts: ‘multimorbidity’ AND ‘general practitioners/general practice’ AND ‘qualitative research’. Once finalised, the Medline search was translated for additional databases: PubMed, Embase (Ovid), PsycINFO (Ovid), Ageline (EBSCOhost), CINAHL (EBSCOhost), Scopus, Web of Science, and the health and medicine subset of ProQuest. All database searches were conducted on 17 September 2018. The Medline version is provided as Additional File 1.

We also performed a general web search using Google and Google Scholar to identify relevant unpublished literature and organisational websites of relevance to primary care and multimorbidity. Multiple different combinations of terms and their synonyms were searched in order to overcome the limitations of web searching; however, we only reviewed the first 50 websites returned per search variant.

We used the following resources to find theses: ProQuest Dissertations & Theses Global, Networked Digital Library of Theses and Dissertations, Theses Canada, British Library’s Electronic Thesis Online Service, TROVE (National Library of Australia), and nzresearch.org.nz. Other reputable sources of grey literature searched include CORE (an open access research aggregator), Grey Literature Report, OpenDOAR, and OpenGrey.

As final measures, we scanned both the online contents pages of the highly relevant *Journal of Comorbidity* (2011–2018) and the reference lists of included studies.

### Eligibility criteria

An eligibility checklist was developed and iteratively tested using a small sample of retrieved citations.

### Types of participants

Studies needed to provide the perspectives of general practitioners. For the purpose of this review ‘general practitioner’ is defined according to The European Definition of General Practice/Family Medicine by WONCA Europe:

… [GPs] are personal doctors, primarily responsible for the provision of comprehensive and continuing care to every individual seeking medical care irrespective of age, sex and illness. They care for individuals in the context of their family, their community, and their culture, always respecting the autonomy of their patients. They recognise they will also have a professional responsibility to their community. In negotiating management plans with their patients, they integrate physical, psychological, social, cultural and existential factors, utilising the knowledge and trust engendered by repeated contacts. General practitioners/family physicians exercise their professional role by promoting health, preventing disease and providing cure, care, or palliation and promoting patient empowerment and self-management… [22].

Studies investigating experiences of GPs as part of a broader group of health professionals (e.g. pharmacists) were included if the first-person contributions of GPs could be independently extracted.

### Phenomena of interest

The phenomena of interest were the perspectives, views, attitudes, or beliefs of general practitioners on the therapeutic management of patients with multimorbidity. Therapeutic management might be pharmacological or non-pharmacological in nature, or involve interventions such as referral, screening, prevention, diagnostic testing, or follow-up [39].

Patients could be 18 years and over with any combination of chronic conditions providing their health care provider considered them a ‘patient with multimorbidity’. Furthermore, we considered an article relevant if multimorbidity was the explicit focus, covered as a subject of interview questions, or emerged as a theme within the study results.

### Context

General or family practices operate differently across countries in terms of practitioner training requirements, funding models, speciality recognition, and the degree to which they serve a gatekeeping role, authorising access to specialty and hospital care. GPs working across significantly different models of general practice will have divergent challenges and experiences which may be difficult to compare. We therefore made a pragmatic decision to limit this review to countries with somewhat similar general practice models, these being Australia, New Zealand, United Kingdom, Ireland, Canada, Netherlands, the Nordic countries, Poland, Portugal, Slovenia, and Spain [40–42].

General practices may be situated within primary care centres where they function as part of a wider health care team, or independently within a private practice. We also included other settings where GPs work such as nursing homes.

### Types of studies

This review considered any study design providing the study reported the verbatim quotes from general practitioners conveying their views, opinions, beliefs, attitudes, and perspectives on the impact of multimorbidity on their clinical practice.

Studies were limited to those in English language. No date restrictions were applied.

### Study selection

Citations were imported into an EndNote X8 Library where duplicate citations were removed. Using an eligibility checklist, one author then screened all titles and abstracts for relevance, moving each to one of three groups created within the Library titled ‘relevant’, ‘irrelevant’, and ‘uncertain’. A second reviewer then screened 20% of the ‘irrelevant’ group citations as a check on first reviewer decision making consistency. Full text articles were obtained for each citation in the ‘relevant’ and ‘uncertain’ groups. Both reviewers then independently reviewed each full text report to determine its relevance. Disagreements between reviewers were discussed until consensus was reached.

### Quality appraisal

The appropriateness of including or excluding qualitative studies in a synthesis based on an appraisal of their quality remains contentious [36, 43]. We chose to conduct a quality appraisal of all included papers in order to gain a richer understanding of the methodological choices within each study. We did not, however, exclude studies judged to be of lower quality as they might still contribute unique themes to the synthesis [36]. One author (RD) used a 10 question qualitative research checklist developed by the Critical Appraisal Skills Programme (CASP) to appraise quality [44]. Quality judgements are provided as Additional file 2.

### Thematic synthesis

PDF versions of all included articles were imported into QSR International’s NVivo 12 qualitative data analysis software.

#### Stage 1. Free coding of study data and findings

One reviewer (RD) performed detailed coding of participants’ verbatim quotes (herein ‘data’) and author ‘findings’ as provided in the Results and Discussion sections of each primary article. This involved reading the relevant parts of text line by line to ensure all concepts were accounted for. This ‘initial coding’ method created ‘tentative and provisional’ codes to be further refined in stage two [45].

#### Stage 2. Developing descriptive themes

Once all data and findings were coded, one reviewer (RD) examined the list of codes for duplicate, overlapping, or redundant codes. Next, each code’s assigned text was reread to check for consistency in coding across the full range of articles. From this process, some further codes were created, and nondescript code names were replaced with more descriptive labels (‘axial coding’) [45]. The resultant list of codes was then sorted by highest to lowest frequency of text assignment to see which codes were predominant and recurrent across the whole set of articles. Using this view as a basis, all codes were then iteratively and hierarchically arranged into conceptually similar or related groups. For example, ‘communication between providers’ was grouped with ‘conflicting advice to patients’ and both put under the broader code ‘Interface of primary care and other sectors.’ These resultant codes become the ‘descriptive themes’ of the review.

#### Stage 3. Developing analytical themes from descriptive themes

All three authors (RD, DM, JT) then discussed the descriptive themes and their relationships, testing new ways of organising and labelling them. From this, more abstract ‘analytical’ themes which ‘go beyond the content of the original studies’ [46] were developed by discussion and consensus between all three reviewers (i.e. triangulation). These analytical themes had to encapsulate and explain the descriptive themes and be richly supported by the data itself.

## Results

### Search strategy and study selection

**Fig. 2:**
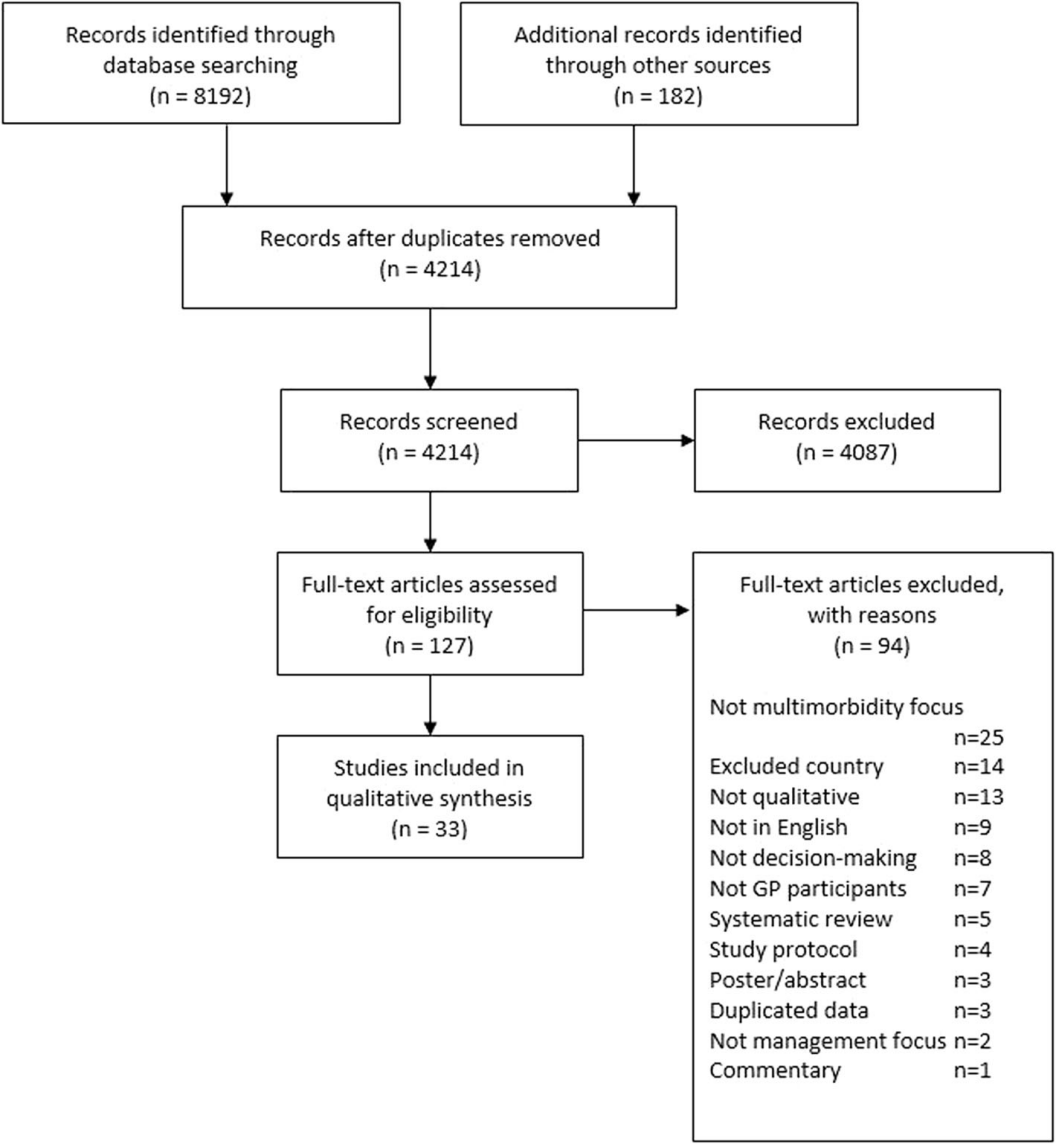
PRISMA flow diagram of article selection decisions

Electronic database and grey literature searches, together with reference list checks, retrieved a total of 8374 citations. This total reduced to 4214 citations once duplicates were removed. After scanning titles and abstracts against the inclusion criteria, 127 citations remained requiring further review by full text article. More detailed full text analysis reduced the set to 33 articles for the final synthesis. Of these, four pairs of studies shared the same data but were retained because they reported on different aspects of it. This process is outlined in Figure 2 as a PRISMA flow diagram [47].

Fourteen individual countries were represented across the 33 studies: Netherlands [48–54], England [55–59], Australia [60–63], Denmark [64–67], Norway [50, 67–69], Ireland [70–72], New Zealand [73–75], Canada [76, 77], Wales [50, 69], Sweden [67, 78], Finland [67], Iceland [67], Portugal [79] and Scotland [80]. Together these studies included a total of 593 unique GP participants (range: 4–74 GPs). The median date across all studies was 2016 (range: 2010–2018).

Studies focused on multimorbidity were usually oriented towards a particular aspect of multimorbidity care. Some used the broad lens of ‘patient management,' [48, 53, 55, 59, 63, 67, 72, 77–80] while others focused on medication management [51, 52, 60, 62, 66, 70, 71, 73, 74]; clinical practice guidelines [49, 58, 61, 68]; GP decision making [50, 69, 75]; care goals [54, 76]; patient self-care [57, 64]; disease management programs [65]; and health service orientation [56].

Further characteristics of the included studies are provided as Table 1.

**Table 1:** Individual study characteristics

Author (Year) and country	No. of GP participants and their characteristics	Patient population and topic focus	Multimorbidity content	Method of data collection	Theoretical framework and form of analysis
Ailabouni (2016a) [73] New Zealand	10 GPs No. of years’ experience prescribing in residential care: 2–32 years	A hypothetical patient with multimorbidity Deprescribing	Focus. Uses a hypothetical patient with multimorbidity to stimulate discussion of multimorbidity	Semi-structured interviews	Theoretical Domain Framework Content analysis
Ailabouni (2016b) [74] New Zealand	10 GPs No. of years’ experience prescribing in residential care: 2–32 years. Gender: 7 males; 3 females	Older patients in residential aged care Deprescribing	Derived theme	Semi-structured interviews	Theoretical Domain Framework Content analysis
Anderson (2017) [60] Australia	32 GPs Mean age: 47 years (range: 28–70 years). Mean time in practice: 18 years (range: 1–50 years). 63.3% full-time. Gender: 18 males; 14 females.	Older patients with multimorbidity in primary care Deprescribing	Focus. Uses a hypothetical patient with multimorbidity to stimulate discussion of multimorbidity	Focus groups	Not stated Thematic analysis using Framework Method
Austad (2016) [68] Norway	25 GPs Not stated	Patients with multimorbidity in general practice Clinical practice guidelines	Focus	Focus groups	Phenomenological approach Systematic text condensation
Blakeman (2012) [55] England, UK	11 GPs Median age: 45 years (range: 30–62 years). Gender: 6 males; 5 females.	Patients with early-stage chronic kidney disease in primary care Patient management	Focus. Section on multimorbidity included in interview guide	Semi-structured interviews	Normalisation Process Theory Deductive analysis using framework
Bower (2011) [56] England, UK	15 GPs ‘A mix of males and females’; ‘significant variation in age and experience among participants’	Patients with multimorbidity in general practice Service organisation; Decision making	Focus	Semi-structured interviews	Not stated Framework analysis
Clyne (2016) [70] Ireland	17 GPs *N* = 14 GPs in practice > 10 years. Gender: 13 males; 4 females	Older patients in primary care Potentially inappropriate prescribing	Derived theme	Semi-structured interviews	Not stated Thematic analysis
Jones (2018) [61] Australia	14 GPs Mean time in practice: 21 years.	Remote Australian Aboriginal patients with complex health problems Clinical practice guidelines	Focus	Semi-structured interviews	Critical theory and a realist paradigm Thematic analysis
Kenning (2013) [57] England, UK	16 GPs Mean time in practice: 17 years. Gender: 7 males; 9 females.	Patients with multimorbidity in general practice Working with patients; self-care	Focus	Semi-structured interviews	Not stated Thematic analysis
Kristensen (2017) [64] Denmark	12 GPs Median time in practice: 16 years (range: 1–41 years). Gender: 6 males, 6 females.	Patients with multimorbidity living in rural, socioeconomically deprived regions Self-care	Focus	Semi-structured interviews	Not stated Systematic text condensation
Kristensen (2018) [65] Denmark	See Kristensen (2017) as duplicate data	Patients with multimorbidity and lowered self-care ability Disease management programs	Focus	Semi-structured interviews	Not stated Systematic text condensation
Kuluski (2013) [76] Canada	4 Family Physicians Time in practice: 3 GPs > 10 years; 1 = 1 year.	Older patients with multimorbidity in primary care Care goals	Focus	Semi-structured interviews	Not stated Thematic analysis
Laue (2016) [69] International but only extracted data and findings for Norway, Wales, and the Netherlands	23 GPs (Norway: *n* = 7; Netherlands: *n* = 6; Wales: *n* = 10) Norway. Time in practice: 2 months-20 years. Netherlands. Time in practice: 3–30 years. Wales. Time in practice: 4–28 years.	Patients with COPD and exacerbations Decision making	Derived theme	Focus groups	Grounded theory Thematic analysis
Laursen (2018) [66] Denmark	14 GPs Mean time in practice: 15 years. Gender: 7 males; 7 females.	Poly-medicated patients with multimorbidity Medication review	Focus	Semi-structured interviews	Phenomenological/hermeneutic orientation Content analysis
Luijks (2012) [48] Netherlands	25 GPs Mean age = 50 years. Mean time in practice: 20 years (range: 2–36 years). Gender: 18 males; 7 females.	Patients with multimorbidity in general practice Patient management	Focus	Focus groups	Realism paradigm Constant comparative analysis
Luijks (2015) [49] Netherlands	See Luijks (2012) as duplicate data	Patients with multimorbidity in general practice Clinical practice guidelines	Focus	Focus groups	Not stated Constant comparative analysis
Mc Namara (2017) [62] Australia	5 GPs (26 health care professionals in total) Not stated	Patients with multimorbidity in general practice Patient management	Focus	Semi-structured interviews	AGS Guiding Principles (cite) used as a framework for analyis Constant comparative analysis
O’Brien (2011) [80] Scotland, UK	15 GPs Gender: 8 males; 7 females	Socioeconomically deprived patients with multimorbidity in general practice Patient management	Focus	Semi-structured interviews	Modified grounded theory approach Constant comparative analysis
Ploeg (2017) [77] Canada	4 Family Physicians Not stated	Older, community dwelling patients with multimorbidity Patient management	Focus	Semi-structured interviews	Thorne’s interpretative description approach Constant comparative analysis
Prazeres (2016) [79] Portugal	74 GPs Mean time in practice: 16 years (range 1–37 years). Gender: 23 males; 51 females	Patients with multimorbidity in general practice Patient management	Focus	Online survey	Not stated Thematic content analysis
Risor (2013) [50] International but only extracted data and findings for Norway, Wales, and the Netherlands	See Laue (2016) as duplicate data	Patients with COPD and exacerbations Decision making	Derived theme	Focus groups	Grounded theory Not stated but ‘line-by-line coding’ used
Sandelowsky (2016) [78] Sweden	59 Primary Care Practitioners Mean age: 46 years (range 28–68 years). Mean time in practice: 14 years (range 1–39 years). Gender: 30 males; 29 females.	Patients with COPD Patient management	Focus	Semi-structured interviews and focus groups	‘Inspired by the grounded theory method’ Constant comparative analysis
Schuling (2012) [51] Netherlands	29 GPs Mean age: 54 years (range 39–65 years). Gender: 27 males; 2 females.	Older patients with multimorbidity in primary care Deprescribing	Focus	Focus groups	Not stated Thematic analysis
Sinnige (2016) [52] Netherlands	12 GPs Mean age: 56 years (range 46–63 years). Mean time in practice: 25 years (range 10–35 years).	Older people with polypharmacy in general practice Medication management; Polypharmacy	Focus of clinical vignettes	Focus groups	Not stated Framework approach
Sinnott (2015) [71] Ireland	20 GPs Length of time qualified: 6 GPs < 10 years; 14 GPs > 10 years.	Patients with multimorbidity in general practice Prescribing decisions	Focus	Semi-structured interviews and chart-stimulated recall	Grounded theory approach Constant comparative analysis
Smith (2010) [72] Ireland	13 GPs Not stated	Patients with multimorbidity in general practice Patient management	Focus	Focus group	Not stated Framework analysis
Solomon (2012) [58] England, UK	8 GPs Not stated	Patients prescribed a statin or a PPI in primary care Clinical practice guidelines; Patient-GP partnership	Derived theme	Semi-structured interviews	Not stated Framework analysis
Sondergaard (2015) [67] Nordic countries: Denmark, Finland, Iceland, Norway, Sweden	62 GPs Not stated	Patients with multimorbidity in general practice Patient management	Focus	Plenary session and short open-ended questionnaires	Not stated Framework analysis
Stanners (2012) [63] Australia	8 GPs Time in practice (range): 20–40 years. Gender: 7 males; 1 female.	Patients with multimorbidity and depression in general practice Patient management	Focus	Semi-structured interviews	Grounded theory Constant comparative analysis
Stokes (2017) [75] New Zealand	12 GPs Not stated	Patients with multimorbidity in general practice Patient management	Focus	Semi-structured interviews	Not stated Thematic analysis
Tonkin-Crine (2015) [59] England, UK	19 GPs Mean age: 46 years (range: 31–60 years). Mean time in practice: 16 years (range: 3–32 years). Gender: 12 males; 7 females.	Patients with advanced chronic kidney disease in primary care Patient management	Derived theme	Semi-structured interviews	Not stated Thematic analysis
van de Pol (2015) [53] Netherlands	20 GPs Mean age: 48 years (range: 32–60 years). Gender: 11 males; 9 females.	Older patients in residential aged care Patient management	Derived theme	Focus groups	Not stated Constant comparative analysis
Vermunt (2018) [54] Netherlands	15 GPs Mean age: 51 years. Mean time in practice: 16 years. Gender: 6 males; 9 females.	Older patients with multimorbidity in general practice Patient management	Focus	Semi-structured interviews	Not stated Thematic analysis

### Quality appraisal

The quality of each of the 33 included studies was considered high as judged by the CASP Critical Appraisal tool for qualitative research. Only two areas were inconsistently reported: consideration of the nature of the researcher/participant relationship and of ethical issues. It is not possible to know if these elements had been considered by researchers and not reported or not considered at all. We have therefore marked these areas with a question mark rather than a ‘No’ response (Additional file 2).

### Thematic synthesis

The thematic analysis of GP experiences of multimorbidity focused on three broad areas established a priori to answer the specific review questions. These were challenges of patient management, strategies for dealing with challenges, and suggestions for improvement.

In looking at inherent challenges, we identified three predominant analytical themes: *Practicing at the bounds of evidence*; *Confronting patient complexity;* and *Intersectoral failures and problematic policy.* Two themes emerged from the data to help our understanding of how GPs manage these challenges: *Prioritising a patient-centred approach to care*; and *Strategies for managing the consultation*. To answer review question three, we extracted GP views on what is needed to help them better serve this patient population. Further illustrative quotes supporting all themes are provided as Additional file 3.

### GP perceptions of challenges in multimorbidity management

#### Theme 1. practising at the bounds of evidence

GPs questioned the applicability of existing therapeutic research to patients with multiple chronic conditions and their awareness of evidential limitations in this context created uncertainty and risk. It also induced a tension between practicing according to guideline recommendations—which might be mandatory within a specific national or regional primary care system—or deviating towards a more patient-centred, less evidence-based approach.

##### The existing evidence base: insufficient, non-generalisable, and potentially unsafe

GPs considered clinical practice guidelines to be oriented around the management of single conditions—an approach aligned with secondary, rather than primary care [49, 51, 57, 62, 65, 67, 68, 72, 74, 75]. This could render guidelines ‘reductionist’ [57, 68] and an inadequate foundation on which to base clinical decisions [49, 50, 58, 67, 70–72]. GPs described guidelines as silent on the cumulative effects of prescribing different medications for concurrent conditions. This left them in the difficult position of having to weigh the potential benefits and risks of each patient’s unique therapeutic cocktail. GPs were therefore operating in the knowledge that treating one problem risked exacerbating or creating new problems for other conditions [49, 51, 57, 60, 65, 67–69, 72, 74, 77, 79].

The problem is that you are trying to weigh up unmeasurable harm quite often against unmeasurable benefit. We are trying to do that in our minds and trying to work it out—Is it more likely to be doing benefit or more likely to harm? The truth is that, in many cases, I don't know [60].

GPs were concerned that following a different guideline for each condition might jeopardise patient safety by driving polypharmacy, overdiagnosis and overtreatment ('… we're poisoning our patients.') [72] This approach also imposed a high burden of treatment on patients putting patient adherence at risk [65].

Guidance on deprescribing medications in the face of problematic polypharmacy was regarded as similarly inadequate [60, 62, 68, 72–74] with one GP describing it as ‘a riskier, less certain, and more cognitively and socially demanding process, with minimal decision support.’ [60] Knowing when and how to deprescribe *preventative* medicines for older patients or those with a poor prognosis was considered particularly important but often challenging as it meant reconciling statistical concepts such as absolute/relative risk reduction, number needed to treat, and time-to-benefit with questions of life expectancy and quality of life [49, 51, 62, 72–74].

With a 40-something year old, the treatment aim is clear...to reduce risk over a long-term period. But for an 80-something year old, it becomes less clear cut [...] What can the patient get out of it, and also, what are the possible side-effects? [49]

GPs attributed the lack of useful and generalisable multimorbidity guidance to the hegemony of the clinical trial methodology with its preferential focus on internal, rather than external validity [49, 51, 68]. They were aware that guideline recommendations were often based on trials that tested therapies using much younger and less complicated patients than those they regularly encountered in their practices [49, 74, 77].

##### Protocol-driven medicine vs clinical judgement

For these reasons, GPs within and across studies frequently differed in the extent to which they viewed adherence to the tenets of evidence-based medicine feasible, or even desirable, in the context of multimorbidity. For some GPs, awareness of the limitations of existing evidence appears to provide a justification for preferencing their own professional autonomy and clinical judgement: ‘[Multimorbidity] gives you a lot of freedom to use your experience and own ideas as a doctor to help the patient’s problem. Otherwise you’d be much more tied to the evidence.…' [49] When this approach didn’t result in negative outcomes, a GP’s self-confidence could be boosted: ‘I think, as you get older, you realize that is not really true because you have done it so many times and they have not had a stroke the next week.’ [60]

Other GPs valued guidelines while viewing consistent adherence to them ‘an impediment’ [49] or ‘a kind of hindrance’ [51] to patient-centred care: ‘Guidelines can only say so much about the disease and nothing about the whole patient.’ [66] Some GPs regarded strict adherence to guidelines as a way to ‘protect their back’ against any professional or legal challenges to their decision making [68]. ‘We could always go back to CARPA and say, “Look, this is how we’re doing it and that’s what’s in the book. So leave us alone.”‘ [61] This kind of ‘defensive medicine’ was also played out when GPs felt it necessary to deviate from guidelines:

When I deviate from the guidelines, I am careful to write my reasons down in the patient record. For instance, if I take a patient off acetylic acid because he developed a stomach ulcer, I write that I am aware of the increased risk of a blood clot. Good record-keeping helps protect me [68].

A further group of GPs said they generally disregarded guidelines due to the overwhelming volume of evidence coming at them, combined with a lack of time or willingness to consult it [60, 74, 78]. This attitude, largely evident in studies from Australia and New Zealand, contrasts with those of GPs in countries where guideline adherence is mandated despite the fact that ‘the map and the terrain simply [do] not match.’ [68] In Norway, for example, GPs describe the pressure to conform to all guidelines as ‘[t]he insecurity that a guideline hell brings,' [68] while in the Netherlands one GP stated ‘I have difficulty not following the guidelines if I don’t have good reasons to do so.’ [51]

##### Clinical uncertainty and perception of risk

In the absence of adequate evidence, GPs had a strong sense of the risks associated with their decision making, [48, 50–54, 57, 59, 60, 62, 63, 66–69, 71, 72, 74, 79] a situation described as ‘doing it without the really significant evidence-based security.’ [60] This could lead GPs to feeling nervous, anxious, or fearful of making mistakes and creating negative consequences for their patients [57, 60, 62, 68, 69, 71–74]. As a result, GPs might adopt one of two mindsets: what Anderson [60] describes as a ‘risk to be reconciled’ or a ‘risk to be avoided’ frame. These orientations could be reinforced by positive or negative past experiences.

Since I’ve started to look at that more globally, the number of medicines I’m prescribing on average for patients in rest homes is about 50% of what I was prescribing a year ago and they aren’t falling off their perch in greater numbers [74].

In avoiding risk, GPs might maintain the status quo or demonstrate ‘clinical inertia’ in decision making [60, 62, 70, 71], especially in the context of deprescribing. This manifested as a reluctance to ‘stir things up’, [72] a focus on removing just the ‘low hanging fruit,’ or waiting for a clear ‘trigger event,’ such as a patient falling, to know when to cease a therapy [60]. The opposite reaction to a sense of risk was to provide, rather than withhold, treatment to patients [66, 68, 69]. This action might be based on the commonly held perception that health care systems rarely criticised GPs for overtreating patients but would take a hard line against GPs who undertreated.

We never get criticized for doing too much. You don’t get in trouble for having initiated unnecessary examinations even if they lead to complications. But you can be sure you’ll get in trouble if you haven’t done enough! We’re much more vulnerable to the entire health care system’s expectation that things must be done. There’s an intense ‘action imperative’ to do more [68].

#### Theme 2. Confronting patient complexity

GPs reserved the term ‘complex’ for a subset of patients whose morbidity burden interacted with advanced age, frailty, or non-medical factors such as social, cultural, or economic context [53, 57, 70, 72]. In fact, any difficulties that impaired patient ability to comprehend the problems, participate in decision making, and self-manage were seen as adding to complexity. This included patient memory loss, cognitive impairment [48, 56, 62, 74, 76], low literacy [61, 80], and patient passivity, lack of motivation or initiative [50, 58, 64, 65, 70, 71]. A low expectation of a patient’s ability to self-manage might escalate into GP feelings of hopelessness [48, 50, 64, 67, 78], or the perception of a patient as ‘difficult.’ [50]

There are a couple of things we encounter such as most patients are 'dead horses'. This does not sound respectful but there are a lot of patients who want to be left alone. We cannot make them understand what we expect from them. Be active, quit smoking, more exercise, loyal to therapy, take their own initiatives [50].

This perception was particularly evident around COPD which some GPs described as a ‘self-inflicted disease’ with low status and low expectations of adherence [50, 67, 78]. ‘You really don’t expect adherence to treatment from someone who has smoked himself to COPD. That’s probably why you don’t refer or treat them.’ [78]

#### Theme 3. Intersectoral failures and problematic policy

GPs described a number of problems in their attempts to share care of patients with multimorbidity with health professionals outside of primary care, chief among them poor communication. This confounded efforts to optimise patient experiences of the health and social care systems and could threaten patient safety. GPs described a sense of professional isolation (the ‘lonely game’ [70]), even demoralisation, when trying to create coordinated, wholistic care for patients in the face of a fragmented system. They were often unsupported in this by policies dictating consultation times and remuneration.

##### The primary-secondary divide

GPs reported a crucial disconnect between primary and secondary care prescribers which often resulted in GP reluctance to deprescribe or modify therapies initiated by specialists, even when they were uncertain of the initial indication [60, 62, 66, 70, 73, 74].

Yeah, look the big doctor in the white coat in the big house on the hill always knows more than the GP especially the house surgeon who might have a brief amount of experience and does what they’re told and one of the issues with this process is, experienced GPs still think that the doctor up the road knows more [74].

Patients may also be unwilling to consider reducing or stopping medications when GP advice went against the perceived higher authority of the specialist: ‘Doctor X said I must never, ever stop that.’ [60] Furthermore, GPs described the information coming to them from specialists as frequently ‘delayed, lost or vague’. [62]

The transition point between hospital and the community setting was considered particularly dangerous for the conveyance of information on patients with multiple conditions. GPs may not be informed of why certain drugs had been added to or removed from the patient’s list, nor whether this change should be considered permanent or temporary [41, 66].

Across several studies, GPs reported wishing to ‘share the onus of responsibility’ of multimorbidity care with specialists, ‘rather than flying solo on it.’ [71] However, endeavours to contact specialists for guidance could be frequently frustrated. According to one GP, this lack of communication had led to ‘[a] collusion of anonymity, which is, you know, this is not my patient, not my patient....’ [72] Not all GPs described a poorly established GP-specialist relationship. GPs in one study regularly contacted renal specialists for advice about CKD and felt buoyed by their availability in the case of a deterioriation [59].

Some GPs perceived specialists as operating in silos with a single disease mindset which could impact significantly on their own workload: ‘If we could stop hospital physicians prescribing single issue medicines for compromised older people, we’d reduce our problems by 50% overnight.’ [74] Specialist prescribing practices might even interfere with the GP’s professional accountability or prescribing autonomy. According to one GP:

I see how patients go into the hospital and have new medications added because the hospital has followed the guidelines. We often have to take responsibility later for having the patients discontinue some meds and we thereby ‘break the rules’. That’s no easy job! But we have to try to see the whole patient [68].

As a result, patient care might become disjointed, with little flow of information and continuity of care between settings [65, 75]. Poorly defined individual roles across sectors led many GPs to attempt to assert professional responsibility for counteracting this fragmented, piecemeal approach by providing holistic, coordinated care. When workload pressures often made this hard to achieve in practice, this could lead to ‘general inaction on multimorbidity’ altogether [62].

##### Issues within primary and community care

GPs raised several areas of difficulty in providing care to patients with multimorbidity living in the community, especially those within residential aged care. Nursing homes presented GPs with a frustrating range of different computer systems and operational policies and procedures, leading them to label the environment as ‘disorganised,’ ‘chaotic,’ ‘ad hoc,’ and ‘deficient in its coordination’ [53, 74]. They described inconsistent and unclear documentation practices, as well as the absence of a shared digital patient record system which could provide data continuity between the nursing home and the GP’s clinic.

You try and find the notes, hard to find. You can’t find the medicine chart, it could be on the rounds somewhere. It’s not computerised, it doesn’t link with our technical notes at the medical practice, so quality just goes down. It shouldn’t be, but at the practice we’ve got the computer, we’ve got light, we don’t have a darkened room in a rest home, and we can actually see what’s going on [74].

GPs specifically mentioned difficulties in dealing with the anonymity, unavailability, and low skill level of nurses in nursing homes: ‘Your first challenge is; you go to the rest home. You try and find a nurse. You can never find one.’ [74] The large number of people involved in providing care in this environment was also seen as problematic as it could lead to ‘… confusion and miscommunication between the staff.’ [53] Overall, GPs found the extra workload, stress and inconvenience in trying to work in nursing home visits around their clinic work as a ‘juggling act’ for which they felt inadequately compensated [74].

Many GPs spoke positively about working with other primary care health professionals, especially pharmacists, to provide team-based care. Pharmacists were seen as particularly important for enabling medication reviews, although a few GPs did not believe pharmacists had sufficient clinical expertise to work independently managing patients with multimorbidity: ‘I am not sure that the pharmacist per se is going to be able to make those decisions. I mean they are probably more clinical decisions.’ [72] This view also extended to practice nurses: ‘... that’s what we spend years doing, is training to make clinical decisions, you know, so you can’t expect nurses to do that, except in a limited way.’ [72]

GPs working in areas of social deprivation reported a different set of local challenges. The social problems they encountered daily had broadened their definition of multimorbidity beyond medical considerations to take in unemployment, poor housing, problems with relationships, and poverty [80]. These GPs spoke of feeling ‘powerless’ to help when they found difficulties in engaging services beyond the clinic in the community to meet their patients’ complex needs [79].

Local clinical systems designed to help GPs with care coordination might also impinge on the management of patients with multimorbidity. GPs working with Aboriginal populations in rural, remote Northern Australia described an inflexible electronic data entry template unable to cater for patient complexity, poorly organised patient data in the electronic health record, and burdensome and inadequately coordinated patient recalls [61]. As one GP said: ‘If I had the time and took the time, I would usually take about an hour [to piece together the story] for people who had chronic health conditions’ [61].

##### Impact of policy on time and workload

GPs reported struggling with the interrelated concerns of inadequate consultation time, insufficient financial remuneration, and increased workload where multimorbidity was concerned [48, 52, 53, 56, 58, 60, 62, 63, 65, 67, 71, 72, 74, 75, 78–80]. Some of these issues appear to stem from existing national or regional health care policies that still view primary care as oriented towards single disease, rather than multiple disease, management.

The foremost topic across studies and countries was the lack of consultation time afforded by health care systems for GPs to provide adequate care for patients with multimorbidity. The fluctuating nature of multimorbidity requires GPs to monitor patients for adverse biophysiological interactions, changes in psychosocial circumstances or preferences for care, as well as any difficulties in communication and care continuity when moving between different health care sectors. This level of vigilance requires more time that the standard consultation time allows.

... [H]ow on earth can you really, in a busy practice, deal with someone with multimorbidity, multi-polypharmacy in a 10-minute consultation? And to be fair to patients you can’t, so you spend longer and therefore your day is longer, and you know, that’s the nature of the job, but it does contribute to an increased workload [72].

Competing demands in multimorbidity care often left GPs just enough time to focus on acute concerns [71]. They therefore tended to put off tackling more time and resource intensive processes such as medication reviews or deprescribing [62]. Opportunities to discuss non-pharmaceutical or behavioural approaches to prevention such as weight loss or exercise are also deprioritised under time pressures [52, 60, 74]. ‘When you see the obese person limping in with a sore throat [you ask]: ‘Do you have a sore throat?’, [and ignore the limp].’ [72]

Dealing with the most pressing medical priorities in the course of a ‘standard’ appointment also limited the GP’s ability to focus on the patient’s concerns [67]: ‘To be honest, you often get that sense [of opening Pandora’s box], and you don’t say anything, because you know you’re at the beginning of the afternoon or whatever.’ [72] This might include their current treatment preferences [62] as well as their longer-term priorities and goals of care [75]. Some GPs believed that this constant ‘…rationing out [of] time’ [80] had a detrimental effect on their performance [67, 72], perhaps even putting patient safety at risk. This concern was evident regardless of GP length of time in practice and clinic location.

Problems with lack of time and extra workload were not helped by remuneration systems which GPs believed provided inadequate compensation for the level of care required by their patients [60, 62, 67, 74, 75]. This view appears to hold sway regardless of whether the primary care model of remuneration is based on fee-for-service, fee-for-performance, capitation, or a mixed model. Any incentives provided were not proportionate to the extra time required for consultation, follow up, and medication review.

Some participants used emotive language in describing the effect of this workload on their resilience, confidence and motivation, especially when patients seemed to make few health gains.

Not worn down, that’s not the right word, but they are difficult to manage because they don’t seem to get any better and then obviously that has a psychological impact probably on the doctor and on the patient [57].

Others used terms such as ‘burn-out,' [79] ‘exhausting,' [80] ‘demoralising,' [80] ‘draining,' [77] ‘overwhelming’ [77] and ‘soul destroying,' [80] or described feeling like a ‘wrung out rag.’ [80] Conversely, a few GPs working in more deprived areas felt ‘energised rather than de-energised’ and emphasized ‘the privilege and rewards’ of supporting ‘complex’ multimorbidity patients [80]. For one GP, there was a need ‘for me to try and find something positive in it for my own sanity and peace of mind and, if not possible, just accept that there’s a group of people whose lives you can’t change, so don’t try.’ [80]

### How GPs manage the challenges of multimorbidity

#### Theme 4. Prioritising a patient-centred approach to care

Across all 33 studies, GPs described the importance of having and maintaining a good relationship or ‘partnership’ with their patients. Many prioritised this relationship above all other aspects of care, perceiving it to bring benefits to the consultation and treatment outcomes, including the amelioration of certain challenges associated with multimorbidity. Firstly, the GP-patient relationship could provide a solid foundation for garnering better knowledge of patients and their specific life circumstances. This enhanced knowledge might then translate into individually tailored care for each patient based on a richer understanding of patient difficulties, treatment preferences, life goals and personal values.

##### Building and safeguarding a continuous patient-GP relationship

GPs prioritised building and maintaining a long-term, continuous therapeutic relationship with their patients with multimorbidity, viewing this relationship as a prime facilitating resource in patient care [48, 50, 51, 55, 56, 58, 60, 63–66, 71, 72, 75, 79, 80]. It enabled GPs to see the patient beyond the level of a presenting illness and could provide insights into aspects of the patient’s social circumstances and individual psychology which might impact on therapeutic acceptance and success. A long-term partnership was also welcomed as a counter against the short consultation times within which GPs are forced to operate. Seeing a patient over a long period of time allows GPs to work at a slower pace, ‘chipping away’ at providing better follow up, monitoring the safety of any medication changes, and gradually introducing self-management skills [56].

So it does make it easier when you do build up that relationship with patients, that you do see the same ones for these conditions, because then you realise, partly you don’t have to deal with it all in one go, these are chronic conditions and you are going to be seeing this patient regularly, they build up that trust with you that they can come out with things that are bothering them, and that very, very frequently happens [56].

A GP's knowledge about a particular patient could serve as a frame of reference, adding a sense of security and confidence to assessment and decisions [69]: ‘I think, if it is somebody who I know, I know their background, what the plan is and where we are heading, I am involved in the care relationship with them, that gives me confidence.’ [60] This knowledge could also provide a dependable ‘baseline of well-being’ [63] with any deviations from this baseline sending up ‘contextual red flags.’ [69] GPs might call this their ‘intuition’ [63, 65] or ‘gut feelings’ [60].

GPs also valued the trust that often came with relational continuity: ‘I think that you need to gain the trust of the patient, and that trust can be gained, I think, by showing interest, by talking with them about the social context.’ [48] This trust could extend both ways with GPs trusting the knowledge patients were able to contribute to the decision-making process: ‘Which drugs do you think are responsible? Patients are mostly right.’ [51]

Preserving the relationship was often deemed so crucial in the management of multimorbidity that GPs might prioritise its safeguarding above communicating difficult information or changing a suboptimal course of therapy [51, 58, 66, 71]. This could lead to trying to please the patient by ‘going down the path of least resistance’ [66] or avoiding discussing life expectancy versus quality of life for fear of affecting the relationship [51]. Conversations around discontinuing preventative medicines were considered particularly risky as GPs worried that patients might perceive them as ‘giving up on the relationship’ [51, 71] or ‘writing them off’ [74].

In those papers describing GP care in socially deprived areas, the therapeutic relationship appeared particularly intense with participants likening the GP role to that of a ‘priest’ or a ‘friend.’ [80] However, other GPs working in the same area were reproving of this level of familiarity, particularly with patients perceived as having ‘entrenched social problems’, ‘chaotic lives’, or concurrent mental and physical conditions. These GPs felt long term interaction might risk patients becoming too dependent on the relationship [50, 80], consuming the GP’s time with little expectation of improvement in situation [50]. These GPs spoke of the need for boundaries or limits between care of the ‘medical’ and of the ‘social’ aspects of a patient’s life.

##### Eliciting patient and caregiver values, goals, and preferences for care decisions

GPs appeared to understand the highly individual nature of patient goals and values, accounting for them in the management plan [48, 49, 54, 58, 59, 63, 64, 69, 71, 73, 74, 76]:

I [need to] get a better complete idea about the background, that is, what’s the priority of this old lady, what’s the priority of this man…. [If] I get a better idea [of the background] this will solve many problems [67].

Eliciting patient goals and preferences could often be an intuitive, rather than systematic, process that once again rested on the foundation of a GP-patient relationship [65]. GPs were particularly attentive to the goals and preferences of older patients and those with significant multimorbidity, understanding that goals could change quickly with fluctuating conditions and as the end of life approached [54]. Optimising quality of life then became the main medical goal.

Decision making was often described as a somewhat shared process with the GP in the role of an advisor: ‘You have to go ‘this is your life, your decision’ and then give them my advice but they have to make the decision for themselves.’ [71] However, not all GPs across the studies expressed the importance of eliciting and prioritising patient goals [51, 59, 62]. Some remained focused on clinical issues—often prevention efforts—stating what they viewed as important without reference to patient preferences. The extent to which GPs involved the patient or family in discussion and decision making was also variable: ‘If it is an important decision, then I’ll involve the family. But with some decisions, the family don’t need to know everything.’ [74] Several GPs believed that some patients preferred to be excluded from decision making processes: ‘I just worry about it myself … rather than imparting a huge amount of knowledge’ [71].

##### Tailoring care to each patient’s unique circumstances and illness experience

GPs described using their knowledge of a patient’s unique circumstances to individualise care, even if that meant deviating from the straight application of a guideline recommendation [61]. Having this ‘whole picture’ at their disposal allowed GPs to be more pragmatic in their approach to management and especially self-care as they weighed up a patient’s capacity to meet the financial, emotional, and physical burden imposed by any care strategy: ‘When you have known people for so many years then you really do not need to ask very much about self-care, because you know their work situation, who they are married to, their children and all these things’ [64].

The understanding that comes with relational continuity led many GPs to express empathy for their patients in their illness experience: ‘I worry that what we increasingly ask people to do is something we have got no experience of ourselves... We’re telling them to do some impressively horrible things’ [80].

#### Theme 5. Strategies for managing the consultation

In addition to focusing on the patient-GP relationship and utilising the knowledge gained of the patient, some GPs described strategies for the consultation that ensured action rather than passivity but which came with built-in insurance against risk for both patient and GP [52, 56, 60, 63, 69, 71, 75]. One such stategy was to offset some of the uncertainty by ‘broadening the loop’ to include other health care professionals in the care of a complex patient [71]. ‘[T]o bounce [ideas] off your colleagues just helps, even if it is just something like ‘what in the name of God am I going to do about this’, it’s really important’ [65].

Another common approach across studies was to first prioritise patient problems within a consultation, then manage them sequentially until the consultation time ran out. The patient’s remaining problems are then deferred for a subsequent consultation [56, 60, 71, 75]. Bower et al. [56] call this priority-based, sequential method for dealing with the multiple issues thrown up by multimorbidity ‘the additive-sequential’ approach.

… If they’ve got several conditions and several conditions need addressing, then you’re limited in what you can do in one consultation slot. You get to know them and maybe next time he might say something like, ‘can you make a double appointment next time?’ So it gives them that little bit longer. Or ask if they can come back; you do what you can within your time, usually go over time and then get them to come back for the rest if they haven’t managed to achieve everything [56].

For GPs, this process could generate a sense of ‘having time,’ alleviating some of the stress associated with inadequate consultation length for complex problems [63]. It could also buy more time to deal with diagnostic or therapeutic uncertainties as the GP has a longer time span in which to observe patients for adverse reactions or therapeutic benefits. Chiefly, however, it could aid to build greater relational continuity and the trust that can come with it [75]. These benefits were regarded as especially useful for the diagnosis of depression in patients with multimorbidity [63] and when educating patients about self-management, as all information need not be imparted in one go [63].

GPs might also use ‘safety netting’ as a risk mitigation strategy within the consultation when uncertain of the best course of action but concerned for patient safety. Here GPs prioritise their own agenda for the consultation over that of the patient [75], advising the patient on symptoms to watch for, and building in contingency plans in case the patient’s condition worsened between consultations. GPs also employed ‘satisficing’ in decision making under conditions of uncertainty [60, 71, 75]. Sinnott et al. [71] define this as ‘settling for chronic disease management that was satisfactory and sufficient, given the particular circumstances of that patient.’ This approach was evident whenever GPs discussed relaxing targets or deviating from guideline recommendations (the ‘ideal’) in order to base care more on patient goals and preferences. ‘I think, not perfectly managed, but managed well enough within that person’s individual parameters.’ [75] Satisficing might allow GPs to factor in patient self-care ability, as well as life expectancy:

I’m not aiming for very tight control — I’m happy if his sugars are running a little higher than normal. I mean he has got cardiac failure as well, his life expectancy isn’t brilliant — so long term I think, I don’t think it’s his type 2 diabetes that’s going to kill him [71].

A further strategy described by GPs was to look for synergies between patient conditions and focus the management plan on treating a common causal pathway. This plan could then be sold to the patient as a solution to more than one of the patient’s problems [75].

#### Theme 6. GP views on what might help

GPs provided a range of suggestions for improving the experience of multimorbidity for themselves and for patients. These fell within four main categories: More evidence and knowledge; Collaboration with other health professionals; Adequate consultation length; and Changed approach to care planning and coordination.

##### More evidence and knowledge

GPs expressed the need for evidence and guidelines in both prescribing and deprescribing for patients with multimorbidity [60, 73, 74], especially for the elderly [51]. They believed that current single disease guidelines would only improve their usefulness for patients with multimorbidity if integrated [57], perhaps via cross-referencing [49], if clinical trials involved patients with multimorbidity [49], or if more GPs became involved in guideline development [58]. GPs requested better reporting of guideline external validity [49] and guidance on how to prioritise recommendations, especially for preventative measures [49]. Some saw merit in shifting the focus from disease-specific outcomes towards more generic and global outcomes of value to the patient as these might have applicability across different conditions [49, 56, 79]. GPs also desire readily accessible clinical decision support tools to help their decision making within a number of challenging areas of care [51, 60, 66, 74]. Their suggestions included action cards developed by clinical pharmacologists that could serve as a ‘go-to-list’ when deprescribing [66] and practical tools for prioritising competing conditions [51, 67].

GPs also stated the need for more and better training and education on delivering patient-centred care for people with multimorbidity [53, 63, 66, 67, 72, 74, 79]. Training could take the form of scheduled ‘knowledge exchange’ meetings with other health professionals such as pharmacists and specialists [71], regular refresher courses, or post-academic courses focused on multimorbidity [49].

##### Collaboration with other health professionals

GPs spoke positively about working in closer collaboration with pharmacists in the planning and delivery of medication reviews and for deprescribing [51, 52, 60, 62, 66, 72, 74].

I think we need to carry out medication reviews, and not miss people out. Sometimes its good to have somebody else look at it, so working together with a pharmacist is a good idea. Because I think two pairs of eyes looking at the same page, often gets better results than one person looking at a patient [74].

They also desired better cross-sectoral collaboration, envisaging better communication and support for both themselves and patients through a multidisciplinary team approach [52, 66, 72, 79].

##### Adequate consultation length

GPs argued the need to ‘create a distinct consultation for multimorbid patients’ [79] by extending appointment length by a reasonable amount to afford more time to spend with patients [72, 80]: ‘… [G]ive at least 30 min consultations for these patients;' [79] ‘… if we had time to have longer consultations with them they would consult us less;' [72] ‘… you know, the ideal thing if you could set aside a 40, 45-min slot for each of your multimorbidity patients, and just you know, do a clinic.’ [72] Beyond this, GPs did not suggest how existing systems, policies, and remuneration models could be modified to make extended consultation times a reality.

##### Changed approach to care planning and coordination

GPs raised the need for care plans borne out of a process ‘sensitized to multimorbidity.’ [56] Such a plan would include patient goals and concerns, as well as individualised management strategies, and serve as a formalised, negotiated, and explicit agreement between the patient and the GP [54, 56, 76]. Ideally it would be available digitally [53]. A further idea was to increase the use of care coordinators in supporting patients to navigate multiple health care sectors and providers [62]. This role, which might be taken by a single GP [72], would work on optimising care plans and creating practical measures for improving care coordination [53].

## Discussion

This review synthesised the first-hand accounts of 593 GPs from 14 countries. It confirms the findings of an earlier systematic review that GPs are challenged by inadequate guidelines and fragmented health care systems built around single disease states [25]. It also builds on these findings by identifying additional themes around GPs’ pragmatic strategies for circumventing or managing these challenges and presenting their own suggestions for change.

The data makes clear that GP views are framed by specific national or regional policy levers impacting at the level of their own practice. These levers might dictate if and how patients are to be referred to services such as Disease Management Programs in Denmark [65] or nephrologists in the United Kingdom [55]. They might stipulate how care provided to nursing home patients will be reimbursed [74] while regulating the evaluation of care quality by linking it to clinician performance incentives [56, 68]. Yet despite important local differences, this synthesis identified commonalities between countries in terms of problems faced and approaches for dealing with them.

GPs continue to perceive the evidence base for multimorbidity care as insufficient and incapable of providing guidance for the clinical questions they most need answering. While uncertainty in the face of undifferentiated clinical and psychosocial problems is not uncommon to the GP [81], manifold knowledge gaps around multimorbidity persist [1]. These start with questions at the micro level of biophysiological mechanisms [82] and extend through to macro considerations of the best interventions [83] or care models [84, 85] for improving patient outcomes. Multiple chronic conditions can also present in unique permutations across individuals, challenging diagnostic certainty, limiting management options and altering the treatment benefit/risk profile [86].

It has been known for some time that clinical practice guidelines which prioritise evidence from randomised controlled trials may lack external validity for patients with multimorbidity, being based on younger and relatively healthier populations [87, 88]. The risks of applying a range of recommendations from single condition guidelines to any individual patient were first raised in 2005 [89] and continue to be reported [90–94]. Furthermore, published guidelines continue to inadequately acknowledge comorbidities [95–98]. It is not surprising, therefore, that GPs across the studies in this review demonstrated a cautiousness in strictly adhering to guideline evidence for patients with multimorbidity. They were not insensitive to the potential for iatrogenic harm, overtreatment with little tangible benefit, and increased patient burden of care [18], using terms such as ‘risk’, ‘insecurity’, ‘anxiety’, and ‘fear’ to describe their decision-making experiences. This insecurity appears to affect both prescribing decisions and questions of when to cease unnecessary or harmful therapies. This perception of a lack of safe deprescribing guidance is confirmed in the research literature [99].

Although GPs may be aware of these evidential limitations, some GPs expressed a preference for adhering to guidelines based on the security they represent, viewing decisions based on one’s own clinical judgement as a riskier enterprise. This tension was particularly notable in places where regulatory bodies have linked remuneration or professional accreditation to the attainment of a range of evidence-based quality indicators. It has been widely suggested that these indicators may be less appropriate for patients with multiple conditions as they do not capture the complexity and dynamism of the multimorbidity experience [100]. Furthermore, the outcomes captured by the indicators may not reflect the priorities of patients themselves [101]. For these reasons, alternative evidence-based quality assessment frameworks for complex patients have been proposed or are in development. These might measure and incentivise continuity of care [102], patient preferences and values [100], or use patient-reported indicators to capture the patient’s care experience and outcomes [103].

Gaps or delays in communication from specialists to GPs and specialist inaccessibility to GPs are important system failures which persist despite technological advances such as the Shared Health Record. These inter-professional communication failures provide the GP with an additional, but avertible source of uncertainty as the specialist’s intentions for a patient must be interpreted to minimise treatment conflicts [104, 105]. The problem is not only felt by GPs. It can also impact negatively on patient self-reported perceptions of their care [21, 106–108].

Policies shaping the organisation of care delivery are also shown to have a disruptive impact on the patient consultation by creating a care context ‘structured and incentivized to support short clinical interactions and disease focused care’ [109]. This approach is not only at odds with the principles of patient-centred care endorsed by health systems (even at the national level), but also with the everyday reality of clinical practice where increased patient complexity requires more, not less time and interaction with the GP. Adopting an approach such as the ‘additive-sequential’ model suggested by Bower [56] could, therefore, be considered a deferment tactic indicative of a workload problem, rather than an effective approach to patient care. Currently GPs in the United Kingdom are facing increasing workloads [42] and the strain of trying to meet the higher volume and intensity of work is said to portend a crisis of GP retention [110]. GPs have attributed their widespread low morale and exhaustion to limited time and resources for dealing with increased patient complexity, together with non-commensurate financial rewards [111]. Lack of time to deal with the problems faced in general practice and high rates of GP psychological stress were recently highlighted as concerns in Australia as well [112, 113]. Together, the many negative terms used to describe multimorbidity across included studies in this review may be telling of more extensive morale and stress issues in this context. Without correction, increasing workloads and rising societal expectations of GPs may threaten the goals of both the Triple and Quadruple Aims [114].

Considering the strong association of multimorbidity with aging populations, we were surprised to identify only two studies focused on GP care provision for residential aged care patients with multimorbidity [53, 74]. Here again GPs conveyed a sense of hopelessness in achieving a reasonable standard of care for their patients due to time pressures, poor communication between care providers, inefficient local systems and policies, and a perception of multimorbidity care as being beyond the skill level of some nurses. Models of primary care and their associated financial incentives also appear to impact on GP satisfaction with their residential aged care duties.

### How GPs perceive their role

Despite the many challenges they confront, GPs see themselves as having an important role in managing patients with multimorbidity. The views conveyed within the data strongly support the general practice ethos of providing holistic care with an emphasis on the biopsychosocial context, including family and community. GP concerns for patient safety and wellbeing were expressed in terms that align well with the concept of the ‘patient-centred consultation’ as operationalised by Stewart [115]. GPs strove to understand the patient’s illness experience; see the whole person in context; find common ground; and enhance the doctor-patient relationship through, for example, compassion and the gaining of trust [115]. The approach described by many GPs in the study also resonates with the Sage Consultation Model of the ‘expert generalist’ which takes an inductive, data driven approach to decision making by combining information from research, patient, and professional to ‘co-create an individualised account of illness’ [116].

Most strikingly, GPs’ attitudes aligned strongly with the WONCA statement that GPs work by ‘utilising the knowledge and trust engendered by repeated contacts’ [22]. This was clear from the importance GP participants placed on relational continuity, appearing to safeguard it in the face of protocolised targets and guidelines. GPs might value the longitudinal interpersonal relationship with a patient for quite pragmatic reasons, including its usefulness as a ‘simple and powerful tool’ for addressing multimorbidity [117]. It could be relied on as an important source of accumulated knowledge of the patient—one existing beyond the limitations of an inflexible or sparsely populated electronic patient record [118]. This knowledge might impart a perceived security to difficult diagnostic decisions by giving some context to any signs of change or deterioration. As Sturmberg states: ‘The ongoing doctor-patient relationship is a necessary a priori to the creation of shared memories, stored as tacit knowledge…’ for this knowledge grants the GP the ability to ‘discriminate between information and noise’ in a patient’s narrative [119]. In addition to this accumulative knowledge benefit, the research evidence supports the importance of interpersonal continuity of care for downstream effects such as decreased mortality [120, 121], reduced emergency visits [122], and hospitalisations [123], and a greater sense of patient satisfaction with care provision [124, 125]. Despite this positive association, there is growing evidence that relational continuity is on the decline [126] or under threat [127]. Prime reasons given for this erosion include a GP workforce shortage, more GPs working part-time, government policies that prioritise access to care over its continuity, and the widening of teams in primary care into ‘super practices’ [128].

### Solutions from GPs

The GP participants offered some solutions for the problems they encountered when asked to do so. Firstly, they desired closer working ties with pharmacists for the purpose of medication reviews. This was seen as a positive way to resolve the dual issues of uncertainty around (de)prescribing and lack of adequate consultation time. Although recent systematic reviews have highlighted the benefits to patients and GPs of integrating pharmacists into general practices [129, 130], the rate of uptake varies across countries. For example, NHS England recently committed resources towards increasing the numbers of clinical pharmacists working in general practices after a successful pilot project [131]. Meanwhile the Australian government funds community pharmacists to conduct medication reviews but pharmacist integration into general practices remains uncommon, despite some small-scale commissioned trials [132, 133] and a Pharmaceutical Society of Australia action plan for 2023 [134]. In comparison to the pharmacist role, the role of the practice nurse/nurse practitioner in supporting general practice was not mentioned. This may be due to the relative ubiquity of the role in the countries studied.

GPs also emphasised the need for longer appointment times for complex patients. Without adequate time, medication reviews, conversations around goals and priorities, and shared decision making remain ‘nice-to-have extras’ [135]. Concerns over lack of adequate consultation time for patients with multimorbidity have been raised elsewhere [111, 136–138], with one study finding 70% of GPs believed longer appointments enhanced patient enablement and reduced their own workload stress [139]. However, appointment length often goes hand-in-hand with models of remuneration to the extent that changing one requires restructuring the other. In Australia, for example, the fee-for-service model rewards GPs more for giving shorter, rather than longer consultations [140], inevitably creating a consultation culture quite different to that of the salaried or capitation general practices. Furthermore, despite GPs wanting more time with complex patients, the evidence supporting longer consultations remains mixed. One Cochrane systematic review of low quality studies found no relationship between length of consultation time and patient satisfaction or health outcomes [141]. Conversely, the 2016 CARE Plus trial could demonstrate a positive impact of longer consultations on patient quality of life and enablement [142].

Finally, GPs proposed a raft of small, somewhat disconnected ideas relating to their current problems with evidence. There was some benefit seen in addressing common risk factors such as obesity, smoking, lack of activity, as well as prioritising the most debilitating disease clusters. More commonly, however, GPs expressed a need for more training and education in how to manage multimorbidity. Although this may be one of the easier multimorbidity challenges to address, the question of how to adequately provide such training to general practitioners remains unresolved [143].

### Implications of these findings

Multimorbidity is ubiquitous and its prevalence is expected to rise as populations age. GPs are at the forefront of care for these patients but there is evidence that they are finding this responsibility a strain. If GPs are struggling to manage these patients, they are at risk of poor personal outcomes such as burnout or loss to the profession which raises concerns for patient safety issues and the sustainability of general practice as a whole. We know that patients have better outcomes in countries with strong primary care [4, 144], and that this may be especially true for patients with multimorbidity [145]. Therefore, as stated by the Royal Australian College of General Practitioners, ‘it is more important than ever to support GPs in their role as health stewards of coordinated patient health care and enhance their ability to provide holistic patient-centred care’ [112].

This study used a qualitative design to focus in on the GP voice to hear what they are telling us works and what doesn’t work. These findings therefore provide some insight into the nature of the problems GPs are facing. Most pressingly, GPs have expressed a need for greater support in providing the generalist care required. This means support from generalisable evidence and from other health professionals, especially those working in other parts of what they consider a fragmented health care system. GPs also require remuneration schemes commensurate with the workload of multimorbidity and the removal of any structural impediments to their ability to forge ongoing relationships with their patients. These concerns may require significant reform of overarching yet antiquated models with considerable system-level support from governments.

### Study strengths and limitations

This synthesis was conducted according to a rigorous methodology that included investigator triangulation for coding and theme derivation and the involvement of researchers from different discipline backgrounds and varied levels of experience with qualitative research. Furthermore, we believe the purposely broad nature of the inclusion criteria and search strategy has ensured that a range of GP perspectives informed the themes.

Restricting the results to countries with similar models of general practice may be seen as both strength and limitation. While it made it easier to meaningfully compare and contrast studies, a future study might involve a cross-country comparison using the included versus the excluded country studies. Furthermore, our included countries may have cultural and socioeconomic differences that influenced findings in unforeseen ways. We were also aware that the primary data sources on which we base our own findings have already been selected and interpreted by other researchers. We do not have access to the full data originally obtained from participants, therefore have no way of knowing if other quotes exist that better support our findings or ably refute them [146]. That said, the richer the data provided in the original papers and the stronger the methodology used, the more confidence we have that authors’ findings (also included in the synthesis) are based on the totality of the data. The quality appraisal process found most of the studies in this review strong on both these attributes.

Similarly, we could only work with what GPs said they do. This means some self-reflections may describe aspirations rather than actual behaviours. The close focus on the topic of multimorbidity may have also led GPs to overestimate the problems or underestimate their own handling of them. Further research may be warranted to determine how generalisable these findings are to a larger number of GPs within the individual health care systems represented here. This might take the form of country-specific cross-sectional studies to verify findings on a larger scale. It might also be useful to ask similar questions of specialists in order to compare their views and experiences of managing patients with multimorbidity to that of general practitioners.

## Conclusions

This paper adds to an understanding of the problems GPs experience in providing frontline care to patients with multimorbidity. The currency of the earliest papers highlights the slow pace at which necessary reforms are being made to health care systems to improve the workplace experiences of GPs and the quality of care received by more complex patients. If we value strong primary care systems we must listen to its practitioners, understand their issues and make concerted efforts to remove barriers to their provision of tailored and patient-centred care. This might also require changes to models of primary care and their systems of remuneration, processes of communication between health sectors, and a focus on multimorbidity education opportunities for GPs and their primary care teams. However, as Salisbury said back in 2013: ‘(T)he time has come to stop just describing the problem of multimorbidity, but to do something about it’ [101].

## Supplementary information

**Additional file 1.** Ovid Medline search strategy.

**Additional file 2.** Appraisal of primary studies according to CASP Qualitative Appraisal tool.

**Additional file 3.** Illustrative quotes supporting derived themes.

## Data Availability

The datasets analysed during the current study are available from the corresponding author on reasonable request.

## Abbreviations

AGS: American Geriatrics Society
CARPA: Central Australian Rural Practitioners Association
CASP: Critical Appraisal Skills Programme
CKD: Chronic Kidney Disease
COPD: Chronic Obstructive Pulmonary Disease
CVA: Cerebrovascular accident
ENTREQ: Enhancing transparency in the reporting of qualitative health research
GP(s): General practitioner(s)
MCC: Multiple chronic conditions
NICE: National Institute for Health and Care Excellence
NZD: New Zealand Dollars
PHO(s): Primary *Health* Organisation(s)
PRISMA: Preferred Reporting Items for Systematic Reviews and Meta-Analyses
QOF: Quality and Outcomes Framework
TIA: Transient Ischaemic Attack
WONCA: World Organization of National Colleges, Academies and Academic Associations of General Practitioners/Family Physicians
