# Supplementary material for: General practitioner strategies for managing patients with multimorbidity: a systematic review and thematic synthesis of qualitative research
Source: BMC Fam Pract. 2020 Jul 1;21:131. doi: 10.1186/s12875-020-01197-8 (PMC7331183; doi:10.1186/s12875-020-01197-8)
Supplement: Supplementary file 1 — Additional file 1. Ovid Medline search strategy. [file 12875_2020_1197_MOESM1_ESM.docx]

# Additional file 1. Ovid Medline search strategy

Datasets included Ovid MEDLINE(R), Epub Ahead of Print, In-Process & Other Non-Indexed Citations, Daily and Versions(R) 1946 to September 14, 2018

| # | Searches |
| --- | --- |
| 1 | Comorbidity/ or Multimorbidity/ or Multiple Chronic Conditions/ |
| 2 | (multimorbid* or "multi-morbid*" or comorbid* or "co-morbid*").tw,kf. |
| 3 | (polymorbid* or poly-morbid* or multipathology or multi-pathology or polipathology or polypathology or pluripathology or poli-pathology or poly-pathology or pluri-pathology).tw,kf. |
| 4 | ((multipl* or "more than" or several or co-occur* or cooccur* or coexist* or co-exist* or concurrent*) adj3 (long-term or longterm or chronic)).tw,kf. |
| 5 | (multidisease* or multi-disease* or multicondition* or multi-condition* or (multiple adj (ill* or disease* or condition* or syndrom* or disorder*))).tw,kf. |
| 6 | Polypharmacy/ |
| 7 | (polypharmac* or poly-pharmac* or polymedicat* or poly-medicat*).tw,kf. |
| 8 | or/1-7 |
| 9 | Primary Health Care/ or General Practice/ or Family Practice/ or General Practitioners/ or Physicians, Family/ or Physicians, Primary Care/ |
| 10 | (primary care or primary healthcare or primary health care or general practice* or general medicine or general practitioner* or GP or GPs or family practice* or family medicine or family practitioner* or family physician*).tw,kf. |
| 11 | or/9-10 |
| 12 | Decision Making/ or Clinical Decision Making/ or Uncertainty/ or Heuristics/ or Judgment/ or Problem Solving/ |
| 13 | Evidence-Based Practice/ or Evidence-Based Medicine/ or Guidelines as Topic/ or Practice Guidelines as Topic/ or Guideline Adherence/ or Clinical Protocols/ or Critical Pathways/ or Algorithms/ or Decision Support Systems, Clinical/ or Decision Support Techniques/ |
| 14 | Health Knowledge, Attitudes, Practice/ or Knowledge/ or Professional Practice/ or Physician's Practice Patterns/ or Professional Competence/ or Clinical Competence/ |
| 15 | Negotiating/ or Consensus/ or Watchful Waiting/ |
| 16 | Patient Care Planning/ or Patient Care Management/ or Patient-Centered Care/ or Patient Preference/ |
| 17 | Inappropriate Prescribing/ or Deprescriptions/ or (deprescrib* or de-prescrib* or ((discontinuing or stopping or ceasing) adj2 medic*)).tw,kf. |
| 18 | (decision* or uncertain* or heuristic* or judg?ment* or problem solv* or reason* or intuit* or gut feeling*).tw,kf. |
| 19 | (evidence based or best evidence or best practice or guideline* or clinical protocol* or critical pathway* or algorithm*).tw,kf. |
| 20 | (knowledg* or competenc*).tw,kf. |
| 21 | (negotiat* or consensus or watchful waiting).tw,kf. |
| 22 | ((patient or multimorbid* or multi-morbid* or comorbid* or co-morbid*) adj1 (manag* or treat* or therap* or prescrib* or prescrip*)).tw,kf. |
| 23 | or/12-22 |
| 24 | "Attitude of Health Personnel"/ or Focus Groups/ or Interviews as Topic/ or Narration/ or "Surveys and Questionnaires"/ or Self Report/ or Grounded Theory/ or Qualitative Research/ or Hermeneutics/ or px.fs. |
| 25 | ((semi-structured or semistructured or unstructured or informal or "in-depth" or indepth or "face-to-face" or structured or guide? or group*) adj3 (discussion* or questionnaire*)).tw,kf. |
| 26 | (interview* or focus group* or diary or diaries or transcrib* or verbatim or field not* or memo? or memoing).tw,kf. |
| 27 | (audiotap* or audio-tap* or audio record* or audiorecord* or tape record* or taperecord* or video*).tw,kf. |
| 28 | ((context* or semantic or content or conversation or discourse* or discurs*) adj2 analys*).tw,kf. |
| 29 | ((narrat* not narrative review) or qualitative* or ethnograph* or fieldwork or (field adj (work or research* or study or studies)) or informant* or phenomenolog* or hermeneutic* or grounded or interpretive* or participant observ* or background observ* or reflective* or reflection* or textual* or open-ended or theme? or thematic* or triangulat* or mixed method*).tw,kf. |
| 30 | ((theoretical or purpos* or cluster) adj2 sampl*).tw,kf. |
| 31 | ((primary care or primary healthcare or primary health care or general practice* or general medicine or general practitioner* or GP or GPs or Family practice* or Family medicine or Family practitioner* or Family physician* or clinician* or provider* or professional*) adj5 (experience or experiences or opinion* or perception* or insight* or perspective* or attitude* or belief* or considerations or awareness or view or views or reflection* or value*)).tw,kf. |
| 32 | or/24-31 |
| 33 | 8 and 11 and 23 and 32 |

**Notes**
/ = search on Medical Subject Headings (MeSH)
tw,kf = search on title and abstract (tw) fields as well as author-assigned keywords field (kf)
px.fs = search on "Psychology" (px) MeSH subheading (fs)
 ⃰ = search for variant word endings
? = allows for zero or one character replacement within a specified word
Adj finds terms next to each other in specified order
Adj1 finds terms next to each but in any order
Adj2 finds terms in any order with one word or less between them
Adj3 finds terms in any order with two words or less between them.
