# Supplementary material for: General practitioner strategies for managing patients with multimorbidity: a systematic review and thematic synthesis of qualitative research
Source: BMC Fam Pract. 2020 Jul 1;21:131. doi: 10.1186/s12875-020-01197-8 (PMC7331183; doi:10.1186/s12875-020-01197-8)
Supplement: Supplementary file 2 — Additional file 2. Appraisal of primary studies according to CASP Qualitative Appraisal tool. [file 12875_2020_1197_MOESM2_ESM.docx]

# Additional file 2. Appraisal of primary studies according to CASP Qualitative Appraisal tool

|  | **Are the results of the review valid?** | | | | | | | **What are the results?** | | | | **Will results help locally?** |  |
| --- | --- | --- | --- | --- | --- | --- | --- | --- | --- | --- | --- | --- | --- |
| **Study** | | Clear aims? | Qualitative methodology appropriate? | Research design appropriate to aims? | Recruitment strategy appropriate to aims? | Data collection appropriate? | Researcher & participant relationship considered? | | Ethical issues considered? | Data analysis rigorous? | Findings clear? | How valuable is the research? | |
| Ailabouni 2016a | | Y | Y | Y | Y | Y | Y | | Y | Y | Y | Y | |
| Ailabouni 2016b | | Y | Y | Y | Y | Y | Y | | Y | Y | Y | Y | |
| Anderson 2017 | | Y | Y | Y | Y | Y | Y | | Y | Y | Y | Y | |
| Austad  2016 | | Y | Y | Y | Y | Y | Y | | Y | Y | Y | Y | |
| Blakeman 2012 | | Y | Y | Y | Y | Y | ? | | Y | Y | Y | Y | |
| Bower 2011 | | Y | Y | Y | Y | Y | ? | | Y | Y | Y | Y | |
| Clyne  2016 | | Y | Y | Y | Y | Y | ? | | Y | Y | Y | Y | |
| Jones  2018 | | Y | Y | Y | Y | Y | Y | | Y | Y | Y | Y | |
| Kenning  2013 | | Y | Y | Y | Y | Y | ? | | Y | Y | Y | Y | |

|  | **Are the results of the review valid?** | | | | | | **What are the results?** | | | **Will results help locally?** |
| --- | --- | --- | --- | --- | --- | --- | --- | --- | --- | --- |
| **Study** | Clear aims? | Qualitative methodology appropriate? | Research design appropriate to aims? | Recruitment strategy appropriate to aims? | Data collection appropriate? | Researcher & participant relationship considered? | Ethical issues considered? | Data analysis rigorous? | Findings clear? | How valuable is the research? |
| Kristensen 2018 | Y | Y | Y | Y | Y | Y | Y | Y | Y | Y |
| Kuluski  2013 | Y | Y | Y | Y | Y | ? | Y | Y | Y | Y |
| Laue 2016 | Y | Y | Y | Y | Y | ? | Y | Y | Y | Y |
| Laursen  2018 | Y | Y | ? | Y | Y | ? | Y | ? | Y | Y |
| Luijks  2012 | Y | Y | Y | Y | Y | Y | Y | Y | Y | Y |
| Luijks 2015 | Y | Y | Y | Y | Y | Y | Y | Y | Y | Y |
| McNamara 2017 | Y | Y | Y | Y | Y | Y | Y | Y | Y | Y |
| O'Brien  2011 | Y | Y | Y | Y | Y | ? | Y | Y | Y | Y |
| Ploeg 2017 | Y | Y | Y | Y | Y | ? | Y | Y | Y | Y |
| Prazeres 2016 | Y | Y | Y | Y | Y | NA | Y | Y | Y | Y |
| Risor  2013 | Y | Y | Y | Y | Y | Y | Y | Y | Y | Y |
| Sandelowsky 2016 | Y | Y | Y | Y | Y | Y | Y | Y | Y | Y |
|  | **Are the results of the review valid?** | | | | | | **What are the results?** | | | **Will results help locally?** |
| **Study** | Clear aims? | Qualitative methodology appropriate? | Research design appropriate to aims? | Recruitment strategy appropriate to aims? | Data collection appropriate? | Researcher & participant relationship considered? | Ethical issues considered? | Data analysis rigorous? | Findings clear? | How valuable is the research? |
| Sinnige  2016 | Y | Y | Y | Y | Y | Y | Y | Y | Y | Y |
| Sinnott  2015 | Y | Y | Y | Y | Y | Y | Y | Y | Y | Y |
| Smith 2010 | Y | Y | Y | Y | Y | Y | ? | Y | Y | Y |
| Solomon 2012 | Y | Y | Y | Y | Y | ? | Y | Y | Y | Y |
| Sondergaard 2015 | Y | Y | Y | Y | Y | ? | ? | Y | Y | Y |
| Stanners  2012 | Y | Y | Y | Y | Y | ? | Y | Y | Y | Y |
| Stokes 2017 | Y | Y | Y | Y | Y | ? | Y | Y | Y | Y |
| Tonkin-Crine 2015 | Y | Y | Y | Y | Y | Y | Y | Y | Y | Y |
| Van de Pol 2015 | Y | Y | Y | Y | Y | Y | Y | Y | Y | Y |
| Vermunt 2018 | Y | Y | Y | Y | Y | Y | ? | Y | Y | Y |
