# Supplementary material for: General practitioner strategies for managing patients with multimorbidity: a systematic review and thematic synthesis of qualitative research
Source: BMC Fam Pract. 2020 Jul 1;21:131. doi: 10.1186/s12875-020-01197-8 (PMC7331183; doi:10.1186/s12875-020-01197-8)
Supplement: Supplementary file 3 — Additional file 3. Illustrative quotes supporting derived themes. [file 12875_2020_1197_MOESM3_ESM.docx]

| Additional file 3: Illustrative quotes supporting derived themes | |
| --- | --- |
| **Themes** | **Illustrative quotes** |
| **Theme 1. Practising at the bounds of evidence** | |
| *The evidence base: insufficient, non-generalisable, and potentially unsafe* | But you don’t have guidelines a lot in the elderly, do you? That’s the hardest thing.^49^  Whereas if you have got one person with diabetes, it’s fairly straightforward to follow the guidelines. People with multiple conditions, there are guidelines for each of them, and it’s impossible…, it’s not beneficial to the patient to stick to 4 guidelines for 4 conditions.^77^  For example, such a guideline for diabetes or hypertension is based on, I don’t know, research on 40-60-year olds ... with mono-morbidity, probably.… And what’s that worth for an 80-year-old patient with multimorbidity? Nothing, in my opinion.^63^  It would be great if the guidelines would mention for whom it doesn't apply, and then I think you'd be shocked at the number of your patients that fall into this category.^63^  If something estimates a risk of death in ﬁve years of 20% or 40 […] it doesn’t really help me (to decide if I should) prescribe a statin or not. If someone’s risk of death is 80% within a year, I wouldn’t prescribe that person a statin.^64^  But when the patients have several diseases, there are too many guideline recommendations. Especially when patients are getting older, how much medicine should you give them?^51^  There are no guidelines yet which can encompass ‘complexity-based medicine’. To grasp how to work with the complexity we confront as GPs requires a massive, theoretical quantum leap. Perhaps in 10–15 years we will realize that all of today’s reductionist guidelines within the natural sciences were wrong and had led us astray.^51^ |
| *Protocol-driven medicine vs clinical judgement* | You sometimes find that they have become over-medicated, but according to the various guidelines, you just have to do it. You can't really treat people like that, right? ^61^  Yes, but if you don’t emphasise the importance or the statistics, then it’s easy to stay in limbo [...] So it’s actually good that one strives as much as possible towards evidence-based ideas over [...] what’s the smart thing to do, or what is the wisest option to reach a good compromise.^63^  With all of the guidelines available, you can use your common sense to say, well, I’d choose this one for this and that reason, that’s easy to justify, or at least I think so. And then the guidelines are definitely not always followed, because common sense in the case of this patient....^63^  It’s also dangerous, doing your own thing, because then it’s just like the way it used to be ... and you do wish that some things were sorted out.^63^  It seems to me as if some of the guidelines’ recommendations are implying: Everybody needs treatment, but so many people just don’t know it yet. We GPs have to counteract this and let our patients know that we don’t think they’ll live any longer or have a better life if we just put them on one additional drug.^51^ |
| *Clinical uncertainty and perception of risk* | Struggling yeah, it’s just not feeling that confident, not feeling that confident about managing one condition, but realising it has an impact on the other one, affecting it adversely. I’m not sure what balance to strike.^56^  You have all this stuff (different guidelines) coming at you and everybody’s saying you could do better, and well maybe we could, in this speciality, but actually, when you’re putting it altogether, maybe we’re not as bad as people like to make out because when you’re sitting there with a lot of stuff, I mean we basically see that balancing huge amounts of information.^49^  ... [L]ike he is very stable on them all, but it does seem like an awful lot.^72^  And that’s the hard thing, whether a little bit of omeprazole, which you’re meant to slowly reduce from 40, to 20, to 10, to stop, and when should that be done?^49^ |
| **Theme 2. Confronting patient complexity** | |
|  | One is my own feelings because when that kind of person comes in my heart sinks a little because I’m thinking what do I do now? I’m thinking what am I going to do this time? Will I be able to cope?^56^  The other problem with a percentage of these patients is that they are, you know, cognitively impaired and it’s very difﬁcult to explain things to them and you have to explain things again and again, and in fact they still get muddled up with their tablets and all the rest of it.^73^  There’s problems with literacy skills. Some of them can’t read they don’t know when their appointments are. They move house all the time either to do with their benefits or are just very peripatetic; sleeping in different places, moving around, don’t always get the mail... It’s difficult to ...offer the same service to these sorts of patients that you offer to somebody who lives in a nice house in [affluent area of Glasgow].^65^  There’s a big problem in that a lot of our patients, their health literacy levels would be extremely low so it is quite challenging to really try and explain what’s going on…. Most people think if they feel fine they’re okay, so they’re more likely to come in when they’re feeling unwell.^55^  The lack of support [...] to help solve many of the problems (which are not organic diseases) that affect the physical well-being of multimorbid patients creates an excessive demand for the use of primary care services. The GP feels powerless to solve social, work and family related problems.^67^ |
| **Theme 3. Intersectoral failures and problematic policy** | |
| *The primary-secondary divide* | So if they’ve seen a cardiologist and were put on a statin, you feel very nervous about stopping it for example. There’s no doubt that you know a specialist assessment, commenced on a speciﬁc drug, you know it does make you reluctant to the change. To change things, yeah, it does have that, it inﬂuences you.^49^  Because I can’t usually just ring up and talk to the (hospital) doctor who wrote the prescription, I have to go through the health information service and sometimes they are more and less helpful cause sometimes they won’t tell you because they are worried about privacy so then you have to go through a process to get it, to have your signature to say are you happy for me to have that information. But that delays things; it means I can’t make a decision now.^64^  The trouble is at the moment there’s no viable model about sharing which would allow us to proceed and obviously because we’ve got two different kinds of systems and not really kind of integrated so it’s a difficult one. (…) The trouble is that specialist medicine doesn’t appreciate a shared model of care really.^77^  …[T]he more complex their prescription, the harder it is for me to do my job, almost as if, the more specialist clinics that people are going to, the tighter the straitjacket I’m on. These elderly people who have a lot of symptomatic illnesses as well, you know, attend me, and I have less and less options.^54^  Precisely when there is multimorbidity, we as GPs have a role of increasing importance. So I think we all need to take responsibility here, and should not have the specialist responsible. […] I am the one who draws up the balance, because in the hospital, there is no generalist.^62^  Basically, older people especially those in rest homes tend to have a lot of co-morbidities, so they are being prescribed things for a lot of conditions. They also have seen lots of specialists who have also prescribed things, and one of the challenges is knowing when you can take control and either stop or reduce doses.^49^  The real difficulty that we have, ok, is that we are basically transcribing hospital prescriptions in a lot of cases onto GMS [General Medical Services Scheme] scripts, ok, and a lot of the time when we get the prescriptions, it’s from an outpatient clinic. It might be in good cases 2 weeks later, in other cases, 5 or 6 months later when we get a letter of explanation for why the changes were made, ok, so do we ignore the prescription until we get an explanation for it?^54^  Some recognition of the problem from secondary care is important, because if people are admitted to hospital, they tend to be put on standard regimes without taking light of their medication and they tend to be looked at in a silos. If they have an MI [myocardial infarction], they’d come out with 40mg of simvastatin and the other side of things won’t be looked at.^49^  If all I had to do was just care about the person’s headache, or their back pain, or their pneumonia, I could just do what I would normally do. But because they are seeing 3 different specialists and because they are on 3 different suites of medicines, you know, I have to cognisant of… You know I’m not complaining about that, it’s just a fact that it does get difficult. You’ve less and less wiggle room you know.^54^  These patients are thrown backwards and forwards between medical specialties in distant hospitals, and it makes it all worse, because they lose contact with general practice . . . These persons, who live here, would be worse off than if they had a single place with stability.^58^  [Patients have] the sense that they’re a collection of organs and, uh, there’s someone that does some work on one part and someone else that does work on another part, and the whole, yeah, that’s your job as a GP, to keep an eye on the whole of the parts.^62^  It’s also very overwhelming to them [older persons with MCC]. They’re on 15 different medications because everybody has been giving them all, but taken all together, it’s just too much.^66^ |
| *Issues within primary and community care* | None of the local rest homes are set up for computerised records which drives me nuts. We are fully computerised (in) general practice these days and when we come to rest homes we go back to this antiquated system of having to handwrite things. Especially, I hate, hate, hate handwriting prescriptions because the room for error goes up exponentially, and then with the multiple faxing of charts.^49^  I still think that this is mainly an issue of knowledge ... The problems we’re seeing in elderly care homes are more complex than they were, say, 15 or 20 years ago. And I just think what’s needed is the expertise [in nurses] to deal with it.^79^  Rest home prescribing doesn’t ﬁt well with my schedule. It is a bit of a juggling act, so I don’t personally like it. If you go to rest homes, some of them are like 15 minutes each side, so doesn’t really ﬁt with me, and even when you say OK I’ll go about 12 or something quickly, they’re eating. They’re in their lunch, so you just end up waiting. Other thing is that you see them at the end of the day, which is again you know, six or something, and then again if you need something, or medication, the pharmacies are closed up.^49^  … So it is not accorded a high, a very high priority and does take place at these extreme ends of the day, and whereas if it’s adequately funded, for you know, 8–8:30 in the morning, and it’s properly funded, and everybody’s there and ready to go, GP’s will go. But while it’s as ad-hoc, as it is, it’s seen as a chore and it’s not well done.^49^  People just don’t have, I mean we’re all busy, and don’t have tons of time. I think we need to carry out medication reviews, and not miss people out. Sometimes its good to have somebody else look at it, so working together with a pharmacist is a good idea. Because I think two pairs of eyes looking at the same page, often gets better results than one person looking at a patient.^49^ |
| *Impact of policy* | You almost need a double appointment for every elderly patient, really, never mind the ones that are complex. It’s a bit like that but we just have to do what we can ....^53^  …[U]sually the true answer, you want the truth? The truth is when you remember, when you are not rushed, then you can do that [deprescribe]. Because that is the next thing on your list, the last thing you would do. One of the last things you would do when it comes to the medication is to cease certain things.^64^  The downside is that it [COPD] increases my workload.... Instead of discussing smoking, referring to spirometry and planning for follow-up, I just write a prescription for antibiotics. Thank you, and goodbye! It goes much faster.^69^  I don’t have that much time.. . for those kind of moral routine stuff, because I already have a lot of acute stuff I got to deal with on the days that I go there [the nursing home].^49^  Lack of time to be able to see the big picture, ending always to work smaller parts at a time and the results are not always good, it leads to forgetfulness, [treatment] redundancies, delays [in diagnosis]....^67^  I always feel there are so many issues! And there are more and more things coming up all the time.... Sleeping problems, a skin problem, and.... It is the GP’s job! Classic!^69^  Then of course you make a rod for your own back because I think by giving people more time and addressing more problems than you should, word gets around, people change to you because a friend recommends you. I've even had people change from doctors within the practice saying that they don't like Dr. So-and-So because he's always in such a hurry and so brusque and efficient, and your heart just sinks because you think, well yes, I will try and do a good job and give more time, but that comes at a cost to me and to my other patients, so you run later and later.^77^  I think New Zealand is in many ways the most difficult [setting to practise GP] because you have two customers scrapping for the same amount of time thinking that they're your exclusive customer. You've got the funding from the Ministry of Health through the PHOs … [who] are not going to pay you if you don't tick [their] boxes. You've got your patient with their A3 list, and both of them want 20 minutes at least of the 15-minute appointment. There's 40 minutes. Two customers fighting for the same time window.^77^ |
| **Multimorbidity management strategies** | |
| **Theme 4. Prioritising a patient-centred approach** | |
| *The continuous patient-GP relationship* | I told her, that her numbers (blood glucose) had worsened. Then she said by herself: ‘Yes, but it is about my (child), who is ill and has just been admitted to the hospital’. Then I said: ‘but yes, I understand’. That is just the advantage of knowing the family... I know their life stories, so I can easily see the whole picture.^57^  We see how they interact with their children. We see how they interact with their neighbours. We sometimes have a much broader view.^62^  I know him quite well, and what his baseline is, so it’s a case of trying to figure out what is the major cause each time he comes in ... we generally try and make a best guess at it.^72^  I suppose when you deal with people for so long you can, you know when they’re different, and you know ... it’s more of an ... intuitive thing, more often than not. You know when they’re not happy….^76^  It’s easier in a face to face consultation, you can judge it much better. Especially when you know them and if they walk the corridor to your room you can tell how good or bad they are.^60^  The fact that we have a personal relationship with our patients plays a part when we discontinue medicine, especially when taking sleeping pills and benzodiazepines, it is obvious that it does, it’s difficult.'^61^  The targets are going to be difficult because patients actually aren’t target driven at all and if… they have the feeling that you are driving them in a direction because the end point is the target, then you actually break down the confidential and therapeutic relationship that you have got with them….^74^ |
| *Patient/carer values, goals, preferences* | I mean, you have to consider how these individuals see their future (…) and how they prefer to shape that future….^80^  What are we trying to achieve with people? Are we trying to make them all live until they’re a hundred and have nursing homes packed with people who ... sitting in nappies all day or are we going to improve their quality of life for the people who are alive now?^73^ |
| *Tailoring care* | I have never liked to pigeonhole patients. It’s completely unrealistic. If we have to treat them equally, we must give different treatments. Twenty per cent of patients might fit into some boxes, but the others don’t. To me, it [stratification] is more constricting than inspiring.58  [G]eneric plans don’t work, you actually have to highly individualise them.^55^  From a medical perspective, I’d say don’t bother… with hemiparesis… but he wanted to, and I know why. It’s because his wife has dementia and he’s her [caregiver].^62^  [Patients] will say, 'Oh, this, this, and this.' I’ll say, 'Well look, we can deal with this and deal with this, but the other, that sounds really important and I don’t want to dismiss it. You will need to make another appointment to come back.' That’s very difficult, because I’m very aware that we charge [NZD] $39 for a consultation. I am very aware that a significant number of people in our area, that’s a big portion of the money that they’re getting that week. It’s not easy. It’s not easy to do that.^77^  The treatment must be planned, individually, based on the patient's functional ability, interests, what he actually manages to follow up on in everyday life, how many activities he can tolerate during a week. The non-pharmacological regimen should not place an additional burden on people already struggling with chronic diseases.^51^ |
| **Theme 5. Strategies for managing the consultation** | |
| *Additive-sequential model etc.* | Sometimes if they've got a whole list of things you have to just sort of divide the list up and say, 'Look, we'll do this today and maybe we can, we need to do something about these things, but then you can come back and we'll do the other thing’….^77^  …[A]nd you don’t have to do it all at once, because you’ll see them again. So I can introduce the idea, and then follow it up.^76^  I think often they've got their agenda of what they want to talk about. You've got your idea that, okay, you want your prescriptions, but I also have to check a number of other things. Trying to focus on what they've actually come in for, which may not be the most urgent thing but is obviously the thing that's worrying them the most, and picking at there's nothing particularly dangerous that you're missing like the ones who at the end of the consult say, 'Oh by the way, I've been having chest pain for the last six weeks.'^77^  The classic example, if somebody has diabetes and also osteoarthritic, you can say to them, 'look if you lose weight then it will take the pressure off your joints, it will make your joint pains easier and you will get into a good cycle where you are actually losing weight, exercising and then maintaining your health.' So there has to be a carrot element to it.^77^ |
